# The geographic distribution of private health insurance in Australia in 2001

**DOI:** 10.1186/1743-8462-6-19

**Published:** 2009-08-17

**Authors:** John Glover, Sarah Tennant, Stephen Duckett

**Affiliations:** 1 Public Health Information Development Unit, University of Adelaide, South Australia, 5005, Australia; 2 Australian Centre for Economic Research on Health, Mayne Medical School Building, University of Queensland, Herston Road, Herston, Queensland, 4006, Australia

## Abstract

**Background:**

Private health insurance has been a major focus of Commonwealth Government health policy for the last decade. Over this period, the Howard government introduced a number of policy changes which impacted on the take up of private health insurance. The most expensive of these was the introduction of the private health insurance rebate in 1997, which had an estimated cost of $3 billion per annum.

**Methods:**

This article uses information on the geographic distribution of the population with private health insurance cover to identify associations between rates of private health insurance cover and socioeconomic status. The geographic analysis is repeated with survey data on expenditure on private health insurance, to provide an estimate of the rebate flowing to different socioeconomic groups.

**Results:**

The analysis highlights the strong association between high rates of private health insurance cover and high socioeconomic status and shows the substantial transfer of funds, under the private health insurance rebate, to those living in areas of highest socioeconomic status, compared with those in areas of lower socioeconomic status, and in particular those in the most disadvantaged areas. The article also provides estimates of private health insurance cover by federal electorate, emphasising the substantial gaps in cover between Liberal Party and Australian Labor Party seats.

**Conclusion:**

The article concludes by discussing implications of the uneven distribution of private health insurance cover across Australia for policy formation. In particular, the study shows that the prevalence of private health insurance is unevenly distributed across Australia, with marked differences in prevalence in rural and urban areas, and substantial differences by socioeconomic status. Policy formation needs to take this into account. Evaluating the potential impact of changes in private health insurance requires more nuanced consideration than has been implied in the rhetoric about private health insurance over the last decade.

## Background

Private health insurance has been a major focus of Commonwealth Government health policy for the last decade. Over the first five years of the Howard government's term in office (a coalition of the conservative Liberal and National Parties, in office from 1996 to 2007), the Commonwealth Government introduced a new policy to increase the prevalence of private health insurance every 18 months. The government's focus on private health insurance was probably stimulated by the sustained decline in the most commonly used measure of private health insurance – the proportion of the population covered for private accommodation in public hospitals – that had fallen from around 50% to around 30% over the previous 10 years [1]. This decline in the headline rate contrasts with the more variable pattern of coverage for accommodation in private hospitals that increased in the late 1980s to a peak of 39% in 1990 followed by a decline to around 33% in 1995 when the series was discontinued [2].

The rhetoric of the Howard government, though, was not cast in terms of private health insurance *per se *but rather in terms of increasing the prevalence of private health insurance to reduce demand on public hospitals; shifting the load from public hospitals to private hospitals. The most significant of the policy changes in terms of take-up of private health insurance was the introduction of lifetime cover taking effect in 1999 [3]. This policy, which encouraged take-up of insurance at or before age 30, led to an increase in health insurance prevalence, with the increase being principally among people who took out policies with front-end deductibles – policies that required them to pay the 'front end' of costs (e.g. the first $500, or $1,000), with insurance covering the tail.

The most expensive policy was the introduction of the private health insurance rebate in 1997, which had an estimated cost of $3 billion per annum [4]. Under this policy, all Australians eligible for Medicare and covered by a health insurance policy including inpatient treatment offered by a registered health fund are eligible for a rebate of 30% of the actual cost of premiums. This policy had relatively little impact on private health insurance prevalence although the policy itself may have been a necessary political precondition for introduction of the life time cover policy.

Given the large expenditures and significant proportion of the population affected, it is surprising that private health insurance policy is a relative data-free zone, especially in terms of data in the public domain. There are two main public data sources on private health insurance: the data published by the Private Health Insurance Administration Council (PHIAC, http://www.phiac.gov.au) [5], which provide a wealth of information on trends in prevalence and patterns of benefits, and data from the irregular (approximately three-yearly) health surveys conducted by the Australian Bureau of Statistics (ABS). In terms of ABS surveys, the long gap between surveys militates against their utility in terms of tracking changes in health insurance.

This article's contribution to health service research is the use of a unique data source on the geographic distribution of private health insurance.

## Methods

An unusual source of data on health insurance was information contained in an answer to a question on notice asked by Senator Jan McLucas in the Senate supplementary budget estimates hearings for 2002–2003, for the Health and Ageing portfolio (reported on 21 November 2002) [6]. Senator McLucas asked for information on the take-up of private health insurance by postcode and federal electorate in Australia. This is the lowest geographic level of prevalence information that has been released about private health insurance. This level of release has not been replicated since then.

The area-based analysis in this article was undertaken using data from the 30% rebate registration database (an administrative collection) in 2001. Due to the once-only registration process – whereby once an individual registers for the 30% rebate (and details of their postcode of residence are available) they remain registered – the collection's suitability for statistical use declines over time. Although data for later periods are not available, it is likely that the socioeconomic patterns described in this report are currently at least as strong, if not stronger, than existed in 2001. For example, insurance cover in 2008 was at the same level as in 2001 (from 44.8% in 2001, cover declined to 43.0% in 2005 and then returned to the 2001 level in 2008) [5]. Further, movement within these overall levels is most likely to be in higher cover for people in areas of higher socioeconomic status, increasing the differentials reported below.

The two questions asked by Senator McLucas were as follows:

1. How many private health insurance contributors, and what proportion of the total, receive the 30% rebate through each of the schemes available for claiming the rebate?

2. With respect to those persons who hold private health insurance which is eligible for the 30% private health insurance rebate and who receive the benefit of the rebate through premium reductions:

a) How many persons are covered by private health insurance by postcode and by federal electorate division?

b) How many contributor units hold private health insurance by postcode and by federal electorate division?

The three schemes referred to in the first question are the Premium Reduction Scheme (91.2% of the rebate paid out through this scheme), the Incentive Payment Scheme (0.2%) and the Tax Offset (8.6%).

Data at the postcode level were analysed to illustrate the characteristics of the population covered by private health insurance. Data were initially allocated to either the capital city or rest of state/territory for each jurisdiction, based on the postcode of the insured (the contributor unit). The 'Other Major Centres' – urban centre of 100,000 or more population at the 2001 Census – were included with the capital city in the same jurisdiction. These were Newcastle and Wollongong in New South Wales; Gold Coast, Townsville-Thuringowa and the Sunshine Coast in Queensland; and Geelong, in Victoria.

Within each of these two major groupings, postcodes were then sorted by socioeconomic status, using the ABS Index of Relative Socio-Economic Disadvantage (IRSD), a summary measure of socioeconomic status derived from the 2001 Census. Postcodes were ranked by their IRSD score then grouped into ten groups (deciles), each of approximately 10% of the population. The proportion of the population with private health insurance was then calculated for each decile. The postcode data were also converted to Statistical Local Area, to allow a correlation analysis (Pearson Product Moment Correlation) to be undertaken against the IRSD.

At the electorate level, percentage coverage was calculated for each federal electorate using electorate populations from the 2001 Census. Each electorate was also allocated the political party of the elected member to allow aggregate proportions to be calculated by political party.

An additional data source was the ABS Household Expenditure Survey 2003–04. Data were purchased on expenditure on private health insurance in five groups (quintiles, each including approximately 20% of the population, based on the IRSD of the Collection District of the contributor unit's address). The proportional distribution of household expenditure on private health insurance in each quintile was applied to the nominal amount of $3 billion (estimated total cost of the private health insurance rebate) to provide an estimate of rebate funds flowing to households in each quintile.

The ABS data included ambulance insurance (where it was separate insurance) and sickness and personal accident insurance. Together, these items represented 13% of the total expenditure (varying from 7% of expenditure in the lowest socioeconomic status areas to 16% in the highest socioeconomic status areas). In addition, the Household Expenditure Survey excludes households in collection districts defined as Very Remote, or Indigenous communities. The impact of this exclusion is most noticeable in the Northern Territory, where the exclusions account for about 23% of the population.

## Results

There were 8,671,106 people who claimed the 30% private health insurance rebate in Australia at 30 June 2001 (46.1% of the Australian population). Of these, 6,468,996 were residents of capital cities and other major urban centres (74.6%) and 2,202,110 were residents of the rest of state/territory areas (25.4%).

Table 1 shows the prevalence of private health insurance by socioeconomic status of the postcode of the insured. Confirming previous studies of prevalence of health insurance in Australia, there is a statistically significant [7] socioeconomic gradient for prevalence, with postcodes in the highest socioeconomic status decile having, on average, almost 70% of residents covered by health insurance compared to residents of the most disadvantaged decile, with a take-up of less than 30%. Not surprisingly, this average figure is also confirmed for postcodes falling within capital cities and major urban centres, with again about 70% of residents in the wealthiest urban postcodes having health insurance compared to fewer than 30% in the most disadvantaged postcodes.

**Table 1:** Private health insurance cover and estimated rebate payments for residents of capital cities and rest of State/Territory^1^, by socioeconomic status, June 2001

Decile	Estimated population with private health insurance cover in			Quintile	Estimated^2 ^rebate ($m) received by people in		
			
	Capital cities	Rest of State	Aust		Capital cities	Rest State	Aust
Highest SES areas	70.8	45.9	68.7	Highest SES areas	679	73	749
2	62.8	44.6	58.2				
3	55.5	45.9	52.0	2	539	163	701
4	52.1	44.8	47.2				
5	48.5	40.6	44.5	3	373	242	615
6	44.0	41.9	43.7				
7	42.3	41.6	41.0	4	231	291	525
8	40.7	37.0	40.3				
9	36.3	38.0	36.4	Lowest SES areas	177	231	410
Lowest SES areas	28.5	27.3	28.1				

**Total**	**48.1**	**40.8**	**46.0**		**2000**	**1000**	**3000**
**Rate ratio^3^**	**2.48**	**1.68**	**2.45**		**3.84**	**0.32**	**1.83**
*Lower 95% C.I.*^4^	*2.47*	*1.68*	*2.44*		*3.35*	*0.25*	*1.55*
*Upper 95% C.I.*^4^	*2.49*	*1.69*	*2.46*		*4.40*	*0.40*	*1.88*

^1^Based on postcode of address of contributor

^2^Estimate based on a total rebate amount of $3 billion: allocation to SES areas based on expenditure on private health insurance (incl. accident insurance), by quintiles of socioeconomic status of area, using the ABS Index of Relative Socio-Economic Disadvantage, 2001

^3^Rate ratio is the ratio of value in Highest SES areas to value in Lowest SES areas

^4^Calculation of 95% confidence intervals (C.I.) based on the comparison of proportions (Berry & Simpson, 1998)

Source: Private health insurance estimates based on data provided by the Senate Community Affairs Legislation Committee (see references). Estimated rebate based on expenditure data purchased from ABS from the Household Expenditure Survey 2003–04

Table 1 also shows the results for non urban centres, including regional cities (of less than 100,000 population) and rural areas. Here we see a quite different pattern of coverage. Again, fewer than 30% of residents in the most disadvantaged decile are covered by private health insurance but, in contrast to the over 70% prevalence in capital cities and other major urban centres, the coverage of private health insurance in the top decile in the rest of the state is much lower, being less than 50%. Although smaller, the difference between these figures is still statistically significant.

There is also strong correlation at the small area level between the distribution of the population with private health insurance cover and socioeconomic disadvantage, as measured by the IRSD; a correlation coefficient of 0.60.

**Figure 1:**
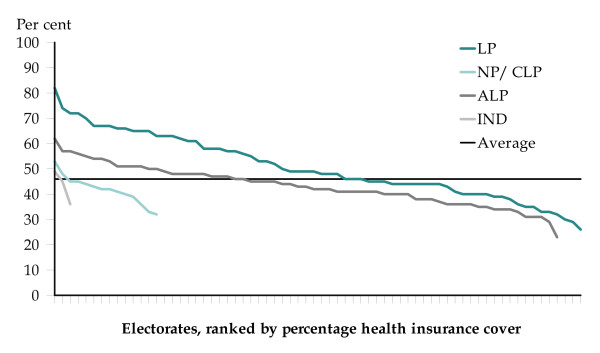
**Private health insurance by federal electorate, Australia, 30 June 2001**. Source: Compiled from data provided by the Senate Community Affairs Legislation Committee [5].

There is a similar distinction in terms of coverage analysed by party affiliation (Figure 1). Within the overall rate of 46.1% of the population covered by private health insurance with hospital cover, seats held by the Liberal Party had an above average coverage of health insurance of 50% [CI: 50.23 ± 0.05] and those held by the other parties and independents had below-average rates: 43% [42.90: ± 0.04] for seats held by the Australian Labor Party; 42% [41.71: ± 0.1] for the National Party; 42% [42.12: ± 0.41] for the Country Liberal Party; and an average of 43% [42.93: ± 0.20] across the three electorates held by independents. These differences are statistically significant.

The range of coverage is from an estimated 23% [22.51: ± 0.28] in the Labor-held seat of Lingiari, in the Northern Territory to over three and a half times (3.6) higher at 82% [81.89: ± 0.50] in the Liberal-held seat of Bradfield, on Sydney's north shore. There is a notable gap of 20.1 percentage points between the Liberal- and Labor-held seats with the highest rates of private health insurance. That is, an estimated 81.9% of the population in the Liberal-held seat of Bradfield were insured in 2001, compared with some 24.6% fewer in (the Labor-held seat of) Jagajaga, with a rate of 61.8%.

Estimates of the allocation of the rebate by socioeconomic status, shown in Table 1), were limited to quintiles (for which the data were available from ABS); these have been aligned with the equivalent deciles. These estimates reflect the marked and statistically significant differences seen in coverage rates for the capital cities and for Australia as a whole; differences related to socioeconomic status.

**Figure 2:**
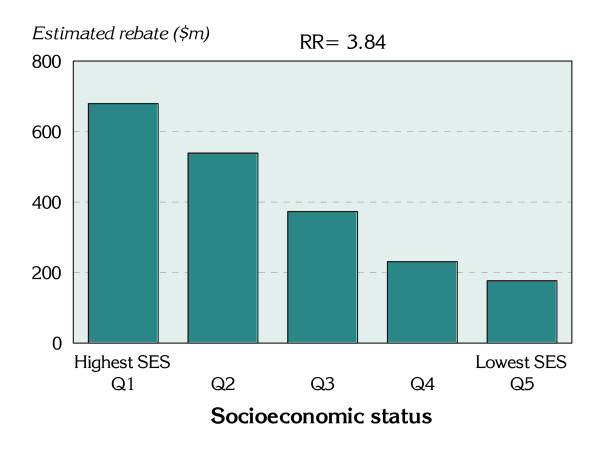
**Estimated rebate payments for people with private health insurance cover, capital cities, Australia, 30 June 2001**.

Notably, for capital cities, the estimated per capita rebate paid to those living in the highest socioeconomic status areas is nearly four times that paid to those in the lowest socioeconomic status areas (a statistically significant rate ratio of 3.84). This represents a substantial transfer of funds to the most well-off, and is a substantially wider gap than exists for private health insurance cover, of 2.48. The difference in these two rate ratios is likely to reflect the larger sums paid for cover, with fewer products purchased with high levels of front-end deductibles, and more products without front-end deductibles, by those in the highest socioeconomic status areas. Also of interest is the strong, continuous gradient evident across the socioeconomic groups, with estimate rebate payments decreasing with each increase in socioeconomic disadvantage (Figure 2).

For the non-urban areas, however, the reverse applies, with estimated rebate payments increasing with increasing disadvantage then declining in the most disadvantage areas. The reasons for this are not clear. One factor contributing to the low estimate in the highest socioeconomic status areas may be the way in which the quintiles are constructed. The areas in the highest socioeconomic status quintile tend to be the towns and other heavily populated areas on the fringes of the capital cities, and the insured population in these areas may be more likely to purchase products with high levels of front-end deductibles, thus reducing the rebate they receive.

## Conclusion

Data presented in the answer to the question on notice did not distinguish the type of private health insurance, and so, for example, no information is available about the take-up of health insurance with high levels of front-end deductibles versus products without front-end deductibles. An individual taking out a policy with front-end deductibles may not intend to use private health insurance as part of a potential hospitalisation, and so again these data cannot be used to infer information about the demand for private hospital accommodation.

Subject to this limitation, there are two clear policy implications of these data. First, although the Labor Prime Minister Rudd (elected in 2007) has made it clear that support for the private sector, such as through the rebate, is here to stay, policy-makers should not think of private health insurance policies in a homogenous way. The marked differences between prevalence in rural and urban areas, and the substantial differences by socioeconomic status, suggest that there is a need for much more nuanced consideration of the implications of private health insurance prevalence.

The second policy implication is that private health insurance has a significant potential to influence the political culture of wealthy (urban) electorates. This might explain the very high importance the Howard Liberal government accorded private health insurance policy, as it was of much higher salience in Liberal-voting electorates.

The implications for policy of the concentration of the insured population in wealthy electorates are difficult to disentangle. To some extent there may be some circularity here. Private hospitals make a business decision to locate private hospitals in areas where there is a greater market for private hospital accommodation. In turn, the market for private hospital accommodation would be driven in part by where people have private health insurance, and, of course, private hospital insurance take-up is more likely if private hospitals are available locally. Policy about private health insurance thus has greater significance for wealthy (urban) areas across Australia and shapes the utilisation of hospital services of the population in these areas. Policies on private health insurance are less important in poorer (and, to a lesser extent, rural) areas across Australia.

This study has shown that the prevalence of private health insurance is unevenly distributed across Australia. Policy formation needs to take this into account. Evaluating the potential impact of changes in private health insurance on public hospitals, or, indeed, given the high correlation between private hospital insurance and ancillary (or general) insurance, developing or evaluating policies on access to allied health and dental services requires more nuanced consideration than previously implied in the rhetoric about private health insurance over the last decade.

## Competing interests

The authors declare that they have no competing interests.

## Authors' contributions

JG obtained the private health insurance data and developed the concept of presenting it to show associations with the socioeconomic status of the population, and to which groups the rebate flows. ST undertook the data analysis. SD wrote sections of the paper dealing with the historical and policy context.
